# Veterinary Medical Society, University of Pennsylvania

**Published:** 1897-11

**Authors:** M. Jacob

**Affiliations:** Secretary


Secretary.

### VETERINARY MEDICAL SOCIETY, UNIVERSITY OF PENNSYLVANIA.

THE first meeting was held Friday evening, October 8, 1897. The forepart of the evening was spent in the election of officers, which resulted as follows: President, G. Chesley, '98; Vice-President, P. Jones, '98; Secretary, M. Jacob, '99; Treasurer, A. Cunningham, '98; Librarian, C. T. Keiter, '99; Executive Committee, L. D. Horner, '98; F. Taylor, '99; L. L. Cheney, '99; L. A. Nolan, 1900.

The election seemed to be the main feature of the evening. Several more students are expected to join the Society in the near future. The programme for the ensuing year promises to be a very instructive one, and hence of great value to its members.

Meeting called to order at 8 o'clock P.M., October 22d. The name of Mr. Hughes was proposed, and he was duly elected to membership.

Mr. Cheney made a report in regard to the examination of the Treasurer's and Secretary's books, and found them to be correct.

Mr. Chesley delivered an inaugural address as President of the Society; it was short, but very enthusiastic, and was highly appreciated by the Society.

It was moved and seconded that a committee of two be appointed by the President to inquire into the matter of prohibiting strangers in the operating-room during operations.

Mr. Spindler then read an extremely beneficial and interesting paper, entitled "The Characteristics of Holstein Cattle."

Mr. Jones reported an interesting case in regard to how electricity had subdued a balky horse.

The programme for the next meeting was read by the Secretary. The Society then adjourned at 9.30 o'clock. M. JACOB,

